# Book Reviews

**Published:** 1873-07-15

**Authors:** 


### Book Reviews.

The Mineral Springs of the United States and Canada, with Analyses and Notes on the prominent Spas of Europe, and a list of Seaside Resorts. By Geo. E. Walton, M.D., etc. New York: D. Appleton & Co. For sale by Jansen, McClurg & Co., Chicago. 12mo., 390 pp.

The author has endeavored in this volume to arrange all the known facts concerning Mineral Waters, in such manner that they shall be readily accessible. As a practical, scientific treatise on the Mineral Springs of the United States and Canada, this little work supplies a want long felt by the profession generally. A comparison of the various mineral waters of this country with those of the most prominent European Spas, shows that, although the Springs as yet developed do not present a complete list of the various classes of waters, still, for the treatment of many diseases we have waters equal to any in the world; and one potent subdivision, the aluminous chalybeates, the author states, are found nowhere but in America of equal strength. Several maps accompany the work, showing the location, means of access, etc., of the various Springs.

The Function of the Eustachian Tube, in its relation to the Renewal and Density of the Air in the Tympanic Cavity, and to the Concavity of the Membrana Tympani. By T. J. Rumbold, M.D. St. Louis: S. W. Book and Publishing Co., 510 Washington avenue.

Opthalmic Contributions. By Geo. Strowbridge, M.D. Including: I. Dermatoid Tumor of the Cornea. II. An Additional Method for the Determination of Astigmation. III. Cyst of the Iris, removal by operation. Philadelphia: Lindsay & Blakiston.

A Guide to Urinary Analyses, for the use of Physicians and Students. By Henry G. Pifford, A.M., M.D., etc. New York: Wm. Wood & Co. Chicago: Jansen, McClurg & Co. 12mo., 90 pp.

Report of Columbia Hospital for Women, and Lying-in Asylum, Washington, D.C. By J. Henry Thompson, A.M., M.D., Washington, D. C.: Government Printing Office.

Normal Ovariotomy. By Robert Beatty, M. D. Rome, Ga.: from the Atlanta Medical and Surgical Journal. Atlanta, Ga.: Herald Publishing Co.

